# Is it the Ideal Time to Start Prescribing *Cannabis* Derivatives to Treat Endometriosis-associated Pain?

**DOI:** 10.1055/s-0042-1749430

**Published:** 2022-06-13

**Authors:** Omero Benedicto Poli-Neto, Jaime Eduardo Cecílio Hallak, Julio Cesar Rosa-e-Silva, José Alexandre de Souza Crippa

**Affiliations:** 1 Gynecological and Obstetrics Department, Ribeirão Preto Medical School, University of São Paulo, Ribeirão Preto, SP, Brazil; 2 Neurosciences and Behavioral Sciences Department, Ribeirão Preto Medical School, University of São Paulo, Ribeirão Preto, SP, Brazil

Endometriosis affects ∼5%-10% of women of reproductive age and is often associated with painful symptoms like dysmenorrhea, dyschezia, dyspareunia, and even non cyclical pain. 1 The disease is diagnosed in at least 20% of women with dysmenorrhea and/or non-menstrual pelvic pain, reaching a prevalence of 50% among adolescents. 2 There is an alignment among international societies 3 4 that the presumed diagnosis of this disease is enough to start clinical treatment. Moreover, there seems to be a consensus that first-line treatment should be hormonal contraceptives since the efficacy is similar to that of surgery but with lower complication rates and costs. 5 However, these drugs are effective in only approximately two-thirds of patients, 6 have limited long-term efficacy, 7 and may occasionally lead to undesirable side effects. Additionally, there are serious limitations in the interpretation of clinical trials. 8 Accordingly, evidence on the best therapeutic regimens has not yet been established. 9 Other clinical options exist, but the cost, side effects, and similarity of results compared with hormonal contraceptives give them limited utility. 10 Thus, due to the persistence of pain, a significant portion of women undergo surgery, which is obviously capable of eliminating visible endometriotic lesions, but not curing the disease. 11 Despite short-term clinical improvement, postoperative recurrence is common, especially if hormone therapy was not initiated. 12

Thus, the clear clinical demand for more effective or lasting options for symptomatic relief, together with an increasing recognition of the participation of the central nervous system in the genesis and/or modulation of chronic endometriosis-associated pain, 13 has aroused growing interest in novel therapeutic modalities. 14 Among these treatments, drugs derived from the *Cannabis sativa* plant, which we will call cannabinoids in the following text, currently seem to be the main topic. In fact, increasing attention has been directed to the potential beneficial effects of these medicines in controlling the symptoms of patients with chronic pain. 15 *Cannabis* contains over a hundred chemical compounds that act on the endocannabinoid system, yet two are rather distinct, delta-9-tetrahydrocannabinol (THC), which is responsible for the psychoactive effects associated with the use of this plant, and cannabidiol (CBD), which does not produce psychomimetic symptoms. 16 Overall, unlike THC, CBD is not addictive or tolerant and has a very favorable safety and adverse effect profiles. At first, it was believed that cannabinoids produced their analgesic effects by the direct activation of specific receptors (CB1 and CB2). However, it is now known that they can reduce pain by interacting with a wide range of cannabinoid, opioid, vanilloid, serotonergic, and anti-inflammatory receptors. 17 Furthermore, preclinical studies have shown that CBD can interfere with the levels of cytokines potentially involved in the pathophysiology of endometriosis-associated pain 18 ; CBD has been shown to decrease the secretion of pro-inflammatory cytokines, including IL-6 and TNF-α, and increase levels of anti-inflammatory cytokines, including IL-10. 19 In addition to these broad potential pain-related mechanisms of action, there is a vast evidence on its anxiolytic, antidepressant, neuroprotective, mood-stabilizing, sleep-modulating effects of cannabinoids, along with many other benefits, 20 which may be useful in the concomitant treatment of non-painful symptoms as the aforementioned comorbidities are also frequent among patients with endometriosis. This makes cannabinoids potentially useful in treating patients with pelvic pain secondary to endometriosis.

A recent Australian national survey found that 13% of women with surgically confirmed endometriosis reported significant positive effects of using *cannabis in natura* both on relieving pain and reducing the use of pharmaceutical drugs as a form of self-medication. However, as the study was not controlled and the investigational products did not have a pharmaceutical grade as a standardized formulation, the conclusions and the reproducibility of findings are limited. 21 Nevertheless, similar findings have been reported in other longitudinal studies. 22 However, at least two meta-analyses focusing on different types of pain 23 24 clarified the limitation of the methodological designs available thus far. Furthermore, they raised a legitimate concern about the significantly higher prevalence of the adverse effects on the nervous system and psychiatric disorders associated with THC use, 24 particularly including psychosis, depressive episodes, and cognitive alterations commonly. More specifically, regarding the treatment of endometriosis-associated pain, two clinical trials registered on the clinicaltrials.gov platform (NCT03875261 and NCT04527003) were retrospectively proposed by researchers from Barcelona and Pennsylvania to assess the effect of cannabinoids on hyperalgesia in women with deep endometriosis, yet both are currently “not yet recruiting.” To the best of our knowledge, in Brazil, we already have a clinical trial in progress and another that will soon start recruiting and is under our responsibility.

Considering the popular saying that “not everything that glitters is gold” there has been a growing concern in the specialized scientific community regarding the increasingly frequent use of *cannabis* or its derivatives for pain relief, despite the potential adverse effects, the lack of robust evidence on benefits and, consequently, the absence of clear recommendations on doses and/or composition to be used. In 2021 the International Association for the Study of Pain (IASP) published a statement position 25 recognizing the legitimacy of the life experience of people who report an improvement in pain following the use of *cannabis* and cannabinoids. Nevertheless, the association made it explicit that it does not endorse the use of cannabinoids until rigorous investigations and robust results clearly show the benefits and harms of its use in humans. The PAIN journal has even allocated an entire collection of 13 scientific articles representing the IASP's Presidential Task Force on *Cannabis* and Cannabinoid Analgesia and calling for high-quality clinical trials to be initiated. Some of the concerns regarding the use of cannabinoids are potential reductions in neurocognitive performance, macrostructural and microstructural brain development, and alterations in brain function secondary to heavy use by adolescents, 26 who have a higher risk of early onset psychosis, 27 and addiction. 28

In Brazil, cannabinoid-based medications are officially approved for use only in patients with refractory epilepsy with a THC concentration <0.2%. These drugs have a very high cost and any use outside the approved indication is off-label. In any case, we have seen a growing supply of *cannabis* -derived products on the market linked to the promise of pain relief. Many serious groups and companies have devoted efforts to drug development, but international quality standards are not followed by all, which poses a health risk as it is impossible to guarantee a high level of quality, adequate pharmacovigilance, and extensive monitoring of adverse reactions. This can also lead to abusive and illegal use.

Nevertheless, the prospect of good results is an encouragement to women with persistent symptoms and professionals who assist them to use cannabinoids, but it is necessary to be aware of the temptation of the premature clinical use of medication. Unfortunately, from a strictly medical and scientific point of view, it is currently impossible to guarantee the efficacy, safety and tolerability of *cannabis* or its derivatives in the treatment of pain symptoms in women with endometriosis. Incentives have been made to disseminate the need for large clinical trials in this domain. To finish, I will restate a part of a text written by Michael Eisenstein 29 which seems to me to be very lucid, sensible and relevant for the situation that we are currently living in: “Unfortunately, if studies such as these are not done—or not done properly—then consumers will be left to fend for themselves in a poorly monitored marketplace. In that scenario, the signal of true clinical benefit would almost certainly be drowned out by the noise from personal anecdotes and the placebo effect, which could jeopardize the future of a potentially valuable medicine.” 29

## References

[1] Eisenberg V H Weil C Chodick G Shalev V Epidemiology of endometriosis: a large population-based database study from a healthcare provider with 2 million members BJOG 2018 125 01 55 62 10.1111/1471-0528.14711 28444957

[2] Janssen E B Rijkers A C Hoppenbrouwers K Meuleman C D'Hooghe T M Prevalence of endometriosis diagnosed by laparoscopy in adolescents with dysmenorrhea or chronic pelvic pain: a systematic review Hum Reprod Update 2013 19 05 570 582 10.1093/humupd/dmt016 23727940

[3] European Society of Human Reproduction and Embryology Dunselman G A Vermeulen N Becker C ESHRE guideline: management of women with endometriosis Hum Reprod 2014 29 03 400 412 10.1093/humrep/det457 24435778

[4] Guideline Committee Kuznetsov L Dworzynski K Davies M Overton C Diagnosis and management of endometriosis: summary of NICE guidance BMJ 2017 358 j3935 10.1136/bmj.j3935 28877898

[5] Chaichian S Kabir A Mehdizadehkashi A Rahmani K Moghimi M Moazzami B Comparing the efficacy of surgery and medical therapy for pain management in endometriosis: a systematic review and meta-analysis Pain Physician 2017 20 03 185 195 28339429

[6] Vercellini P Buggio L Frattaruolo M P Borghi A Dridi D Somigliana E Medical treatment of endometriosis-related pain Best Pract Res Clin Obstet Gynaecol 2018 51 68 91 10.1016/j.bpobgyn.2018.01.015 29530425

[7] Surrey E S Soliman A M Johns B Vora J B Taylor H S Agarwal S K Real-world characterization of women with diagnosed endometriosis initiating therapy with elagolix using a US Claims Database Clinicoecon Outcomes Res 2020 12 473 479 10.2147/CEOR.S264905 32922052 PMC7456657

[8] Capraş R D Urda-Cîmpean A E Bolboacă S D Is scientific medical literature related to endometriosis treatment evidence-based? A systematic review on methodological quality of randomized clinical trials Medicina (Kaunas) 2019 55 07 E372 10.3390/medicina55070372 PMC6681304 31311075

[9] Brown J Crawford T J Datta S Prentice A Oral contraceptives for pain associated with endometriosis Cochrane Database Syst Rev 2018 5 05 CD001019 10.1002/14651858.cd001019.pub3 29786828 PMC6494634

[10] Donnez J Dolmans M M endometriosis and medical therapy: from progestogens to progesterone resistance to GnRH antagonists: a review J Clin Med 2021 10 05 1085 10.3390/jcm10051085 33807739 PMC7961981

[11] Working group of ESGE, ESHRE, and WES Keckstein J Becker C M Canis M Recommendations for the surgical treatment of endometriosis. Part 2: deep endometriosis Hum Reprod Open 2020 2020 01 a002 10.1093/hropen/hoaa002 PMC7013143 32064361

[12] As-Sanie S Till S R Schrepf A D Incidence and predictors of persistent pelvic pain following hysterectomy in women with chronic pelvic pain Am J Obstet Gynecol 2021 225 05 5680 5.68E13 10.1016/j.ajog.2021.08.038 PMC9297195 34464585

[13] Coxon L Horne A W Vincent K Pathophysiology of endometriosis-associated pain: A review of pelvic and central nervous system mechanisms Best Pract Res Clin Obstet Gynaecol 2018 51 53 67 10.1016/j.bpobgyn.2018.01.014 29525437

[14] Carey E T Till S R As-Sanie S Pharmacological management of chronic pelvic pain in women Drugs 2017 77 03 285 301 10.1007/s40265-016-0687-8 28074359

[15] Romero-Sandoval E A Fincham J E Kolano A L Sharpe B N Alvarado-Vázquez P A Cannabis for chronic pain: challenges and considerations Pharmacotherapy 2018 38 06 651 662 10.1002/phar.2115 29637590

[16] Mackie K Cannabinoid receptors: where they are and what they do J Neuroendocrinol 2008 20 01 10 14 10.1111/j.1365-2826.2008.01671.x 18426493

[17] Vučković S Srebro D Vujović K S Vučetić Č Prostran M Cannabinoids and pain: new insights from old molecules Front Pharmacol 2018 9 1259 10.3389/fphar.2018.01259 30542280 PMC6277878

[18] Malvezzi H Hernandes C Piccinato C A Podgaec S Interleukin in endometriosis-associated infertility-pelvic pain: systematic review and meta-analysis Reproduction 2019 158 01 1 12 10.1530/REP-18-0618 30933927

[19] Nichols J M Kaplan B LF Immune responses regulated by cannabidiol Cannabis Cannabinoid Res 2020 5 01 12 31 10.1089/can.2018.0073 32322673 PMC7173676

[20] Crippa J A Guimarães F S Campos A C Zuardi A W Translational investigation of the therapeutic potential of cannabidiol (CBD): toward a new age Front Immunol 2018 9 2009 10.3389/fimmu.2018.02009 30298064 PMC6161644

[21] Sinclair J Smith C A Abbott J Chalmers K J Pate D W Armour M Cannabis use, a self-management strategy among australian women with endometriosis: results from a National Online Survey J Obstet Gynaecol Can 2020 42 03 256 261 10.1016/j.jogc.2019.08.033 31722852

[22] Liang A L Gingher E L Coleman J S Medical cannabis for gynecologic pain conditions: a systematic review Obstet Gynecol 2022 139 02 287 296 10.1097/AOG.0000000000004656 35104069

[23] Chang Y Zhu M Vannabouathong C Mundi R Chou R S Bhandari M Medical cannabis for chronic noncancer pain: a systematic review of health care recommendations Pain Res Manag 2021 2021 8.857948E6 10.1155/2021/8857948 PMC7878090 33613794

[24] Mücke M Phillips T Radbruch L Petzke F Häuser W Cannabis-based medicines for chronic neuropathic pain in adults Cochrane Database Syst Rev 2018 3 CD012182 10.1002/14651858.CD012182.pub2 29513392 PMC6494210

[25] IASP Presidential Task Force on Cannabis and Cannabinoid Analgesia. International Association for the Study of Pain Presidential Task Force on Cannabis and Cannabinoid Analgesia position statement Pain 2021 162 01 S1 S2 10.1097/j.pain.0000000000002265 33729207

[26] Jacobus J Tapert S F Effects of cannabis on the adolescent brain Curr Pharm Des 2014 20 13 2186 2193 10.2174/13816128113199990426 23829363 PMC3930618

[27] Pardo M Matalí J L Sivoli J Early onset psychosis and cannabis use: Prevalence, clinical presentation and influence of daily use Asian J Psychiatr 2021 62 102714 10.1016/j.ajp.2021.102714 34090251

[28] Rup J Freeman T P Perlman C Hammond D Cannabis and mental health: Prevalence of use and modes of cannabis administration by mental health status Addict Behav 2021 121 106991 10.1016/j.addbeh.2021.106991 34087766

[29] Eisenstein M The reality behind cannabidiol's medical hype Nature 2019 572(7771):S2 S4 10.1038/d41586-019-02524-5

